# The Cut‐Sort‐Group algorithm for efficient delivery of collimated step‐and‐shoot proton arc therapy

**DOI:** 10.1002/mp.17889

**Published:** 2025-05-19

**Authors:** Karsten K. Wake, Laura C. Bennett, Blake R. Smith, Wesley S. Culberson, Daniel E. Hyer, Ryan T. Flynn, Kaustubh A. Patwardhan, Nicholas P. Nelson, Patrick M. Hill

**Affiliations:** ^1^ Department of Medical Physics School of Medicine and Public Health University of Wisconsin—Madison Madison Wisconsin USA; ^2^ Roy J. Carver Department of Biomedical Engineering University of Iowa 5601 Sesamans Center for the Engineering Arts and Sciences Iowa City Iowa USA; ^3^ Department of Radiation Oncology University of Iowa Iowa City Iowa USA; ^4^ Department of Radiation Oncology University of Utah Salt Lake City, UT USA; ^5^ Department of Human Oncology School of Medicine and Public Health University of Wisconsin—Madison Madison Wisconsin USA

**Keywords:** collimation, dynamic collimation, Dynamic Collimation System (DCS), Pencil Beam Scanning (PBS), proton arc therapy, treatment delivery time

## Abstract

**Background:**

Proton arc therapy is an emerging technology offering considerably more geometric flexibility than traditional multi‐field treatments, thereby enhancing potential for more conformal proton treatments. The Dynamic Collimation System (DCS) offers energy‐specific collimation during pencil beam scanning to further improve target conformity and reduce dose to normal tissues. Collimation with the DCS during arc delivery is referred to as dynamically collimated proton arc therapy (DC‐PAT). The time required for energy switching, gantry movement during step‐and‐shoot arc delivery, and trimmer movement associated with dynamic collimation necessitates careful planning to create DC‐PAT plans efficient enough to fit within a typical clinical workflow.

**Purpose:**

To demonstrate a post‐processing algorithm to improve the delivery efficiency of DC‐PAT plans while maintaining plan quality.

**Methods:**

A genetic optimizer was used to create baseline DC‐PAT plans for three intracranial cases. These plans were then modified in the post‐processing stage with the Cut‐Sort‐Group (CSG) algorithm. Specifically, each plan was modified through low‐weight control point removal (“Cut”), a novel approach to energy layer sorting (“Sort”), and efficient DCS‐trimmer reconfiguration (“Group”). The components of CSG were evaluated individually and in combination for changes in efficiency, plan quality, and robustness when compared to baseline plans.

**Results:**

After applying the CSG algorithm, the beam delivery time (BDT) for the three patients was reduced to between 10 and 14 min, more than 64% faster than the reference baseline plans. These efficiency gains were achieved with minimal impact on plan quality. The dose coverage to the PTV of the CSG‐derived plans was comparable to the baseline plans for each patient, with the PTV D_2%_ remaining under 10% of the prescription and a Homogeneity Index (HI) ranging from 0.09 and 0.12. Dose to non‐target structures and overall plan robustness was also minimally impacted by the implementation of the CSG algorithm.

**Conclusions:**

The CSG algorithm demonstrates a relatively simple approach to modifying step‐and‐shoot proton arc therapy plans to be more efficient in the post‐processing stage regardless of the treatment planning system or optimization algorithm used to generate the initial plans and with minimal impact on plan quality. The overall BDT was reduced to just over 10 min, approaching plans produced using other advanced optimization algorithms in previous investigations, and fast enough for potential clinical implementation.

## 1 INTRODUCTION

Pencil beam scanning (PBS) proton therapy uses a magnetically scanned proton beam to traverse predetermined beam spot positions, facilitating precise dose delivery to a target. This, when coupled with definable spot energy and spot weighting, enables proton dose modulation in three dimensions, a technique known as intensity‐modulated proton therapy (IMPT).^1^, ^2^ Traditionally, IMPT treatments have employed a multi‐field approach, wherein only a limited number of beams composed of numerous energy layers irradiate the patient from a few (< 5) directions. However, proton arc therapy (PAT), initially proposed in 1997 with passive scattering techniques,^3^, ^4^ has garnered renewed interest in the last decade for use with PBS.^5^ Treatment planning studies comparing PAT to multi‐field IMPT have underscored potential advantages of PAT, including heightened dose gradients, enhanced target conformity, reduced dose to organs‐at‐risk (OARs), improved linear energy transfer (LET) control.^5^, ^6^, ^7^, ^8^, ^9^, ^10^, ^11^, ^12^

The extent of conformity achievable in PBS is partially constrained by the beam spot size of the proton system, defined as the standard deviation of the distribution of in‐air proton fluence measured at the isocenter.^13^ To combat this, the Dynamic Collimation System (DCS) uses two pairs of sliding nickel trimmer bars to provide unique aperture shapes at each energy layer on a per‐beamlet basis, effectively reducing the spot size and lateral penumbra.^14^, ^15^ A functional DCS prototype has been integrated with the IBA ProteusPLUS proton system at the Miami Cancer Institute, and was most recently used to deliver DCS‐collimated treatment plans for several patients to validate a patient‐specific quality assurance process.^16^ Energy‐specific collimation has been demonstrated to enhance conformity for complex treatment volumes, particularly in brain and head and neck cases, and has shown reduced mean dose to nearby OARs in treatment planning studies.^17^, ^18^

Aspects of both dynamic collimation and step‐and‐shoot PAT lead to increased beam delivery time (BDT), hindering progress toward clinical implementation. Several research groups have incorporated different strategies in their PAT plan optimization to reduce the number of low‐to‐high energy switches, which are known to take longer than high‐to‐low transitions (e.g., approximately 5.5 s compared to 0.8 s on the IBA Proteus ONE system^19^), aiming to craft more efficient plans while minimizing compromises in plan quality. These strategies include energy layer redistribution,^20^ removal of low‐weight energy layers,^21^, ^22^ and direct penalization of low‐to‐high transitions in the objective function.^23^, ^24^, ^25^ An investigation of dynamically‐collimated proton arc therapy (DC‐PAT) demonstrated a potential for superior target conformity and healthy tissue sparing compared to multi‐field IMPT plans.^26^ However, adding collimation to PAT contributes to even higher BDT due to additional time required for collimators to translate to planned set points during the treatment delivery. To minimize the additional treatment time needed to deliver DCS‐collimated fields, an ant colony optimization algorithm for obtaining more efficient trimmer sequencing was developed, as presented in the work by Smith et al.^27^

In this work, we explore the feasibility of obtaining more efficient proton arc plans by applying the proposed Cut‐Sort‐Group (CSG) algorithm to modify an existing plan optimized solely for dose objectives. The CSG algorithm features three components. The “Cut” component eliminates low‐weight control points. The “Sort” component employs a novel energy sorting algorithm to reduce energy switching time. The “Group” component uses ant colony optimization to group collimated spots into shared trimmer configurations, thereby reducing trimmer motion time. This approach represents a relatively straightforward method that utilizes post‐processing techniques, independent of the treatment planning system, to generate DC‐PAT plans that maintain high plan quality while being sufficiently efficient for clinical implementation.

## 2 MATERIALS AND METHODS

### 2.1 Treatment planning

An in‐house MATLAB‐based treatment planning system was used to create step‐and‐shoot arc plans for cranial cases of three patients (referred to as patients A‐C). These datasets were used in a previous DCS study^28^ and were obtained through an institutional review board‐approved research protocol and agreement. Each single‐arc plan consisted of 129 equally spaced gantry positions over a single 360° arc (clockwise from −180° to +180°), achieving a resolution of 2.8 degrees. At each gantry position, only one energy layer was used, selected through a genetic optimization algorithm as described by Smith et al.^26^ Spots were uniformly spaced laterally by 3 mm to cover the target volume with an additional 6 mm expansion. Spots located outside of the target volume were trimmed with DCS collimators with a 0 mm offset, meaning the medial edge of the trimmer bars was placed along the central axis of each collimated beam spot. Spot weights were optimized, balancing target coverage with the sparing of nearby OARs.

Dose calculations were performed using Astroid (.decimal, Sanford, FL), an FDA‐cleared treatment planning system, on a 3 × 3 × 3 mm^3^ dose grid.^29^ Each gantry position in this context represents a “control point,” which here encompasses all beam characteristics, including the single selected energy, spot weights, spot positions, collimator configurations, and gantry angle. Importantly, in our use, a “control point” at each gantry position includes multiple trimmer configurations and multiple spots, each with unique monitor unit (MU) weights, delivering a unique configuration of the beam. This terminology may differ from conventional radiotherapy parlance, where each distinct spot, energy, or trimmer setting could be considered its own control point.

**FIGURE 1:**
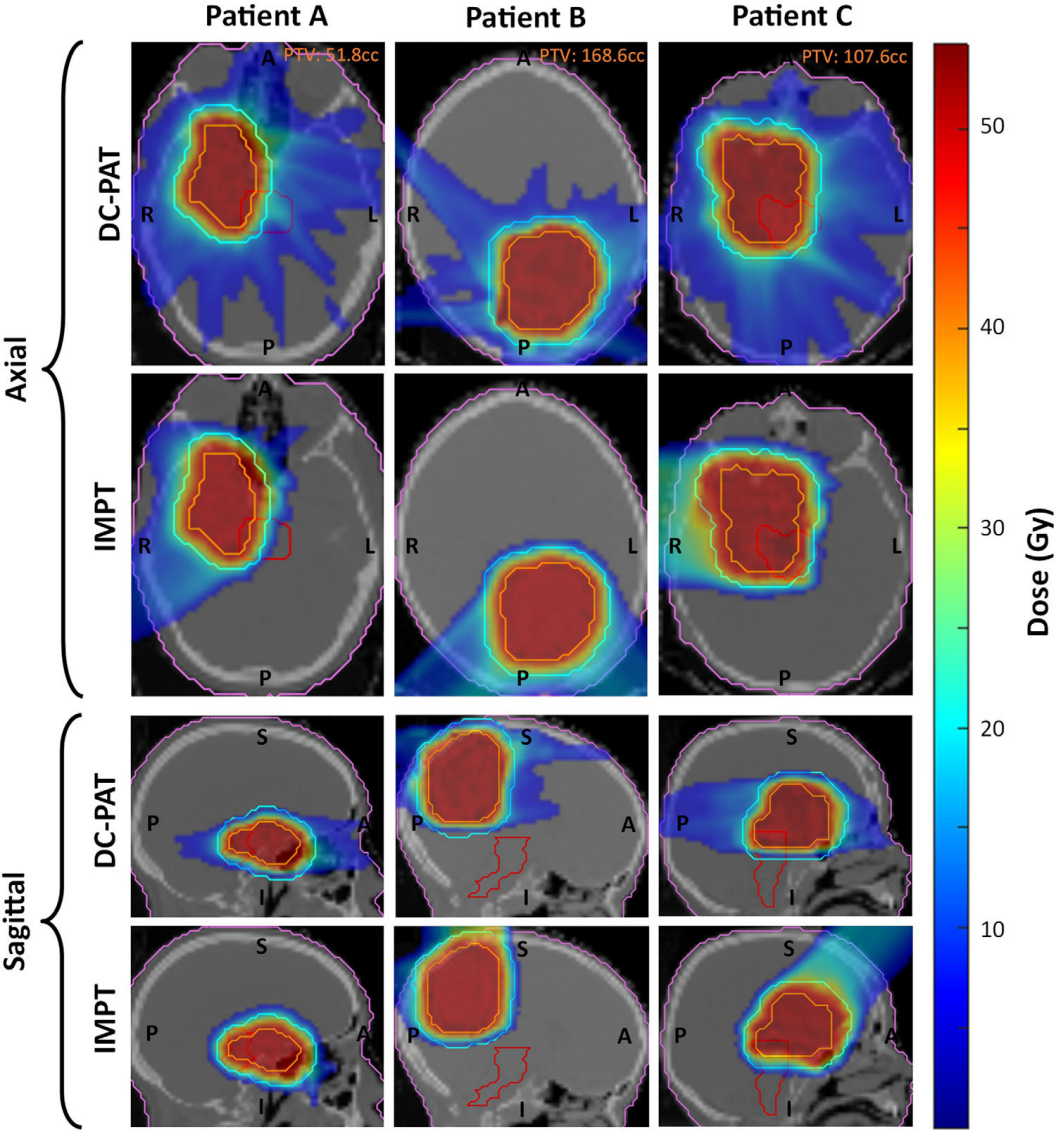
Axial and sagittal dose profiles of baseline dynamically collimated arc (DC‐PAT) and IMPT plans for patient A, B, and C. The PTV, a 10 mm rind surrounding the PTV, brainstem, and head contours are indicated by orange, teal, red, and purple lines, respectively. A dose color wash is displayed from 10% to 110% of prescription dose.

Due to spot‐weight optimization potentially yielding very low (near zero) weights, all spots with prescribed MUs less than 0.025 were excluded from the plans. Consequently, control points containing only these spots were also omitted, reducing the plans originally designed with 129 control points down to 115, 109, and 107 for patient A, B, and C, respectively. These are designated as baseline plans for each patient with sagittal and axial dose distributions plotted in Figure 1. All plans were normalized to D_95% _= 100% of the 50 Gy prescription to ensure fairness in comparison.

A standard uncollimated three‐field IMPT plan was generated for each patient to serve as a comparison to DC‐PAT. Beam arrangements were chosen to balance minimizing surface‐to‐target distance and end‐ranging into critical OARs. The selected gantry (G) and couch (C) angles were as follows: Patient A {G230°:C‐15°, G270°:C55°, G300°:C‐30°}, Patient B {G225°:C0°, G135°:C0°, G90°:C‐90°}, and Patient C {G270°:C0°, G270°:C‐30°, G310°:C90°}. Spot weights were optimized to achieve near‐equivalent target coverage to that of the arc plans, ensuring a fair comparison.

### 2.2 Delivery time estimate

The calculation of BDT is broken into four categories: gantry movement, spot delivery, energy switching, and collimation. Gantry movement time is calculated assuming a step‐and‐shoot arc delivery with the IBA ProteusPLUS, with a max gantry velocity of 6 degrees/s and acceleration of 0.6 degrees/s^2^. Analysis of log files from DCS‐collimated fields delivered on the ProteusPLUS^30^ revealed a linear relationship between spot delivery duration and spot weight, characterized by a slope of 6.6 ms/MU and an R‐squared value of 0.9995. Consequently, the time to deliver a specific spot is calculated by multiplying the spot weight by 6.6 ms/MU and adding an additional 2.2 ms for slew time to allow for magnetic scanning between spot locations. For energy switching, high‐to‐low energy transitions are estimated to take 0.8 s, while low‐to‐high transitions require 6 s.^19^, ^31^ To accommodate movements of the DCS trimmer bars during collimation, additional slew time is programmed into the IBA delivery system, varying with the distance the trimmers must travel between configurations. This relationship between required time and trimmer travel distance, derived from several DCS‐collimated field log files, has been modeled using a second‐order polynomial (R‐squared = 0.9922), serving as a predictive model for the expected collimation penalty in a DCS‐collimated plan.

**FIGURE 2:**
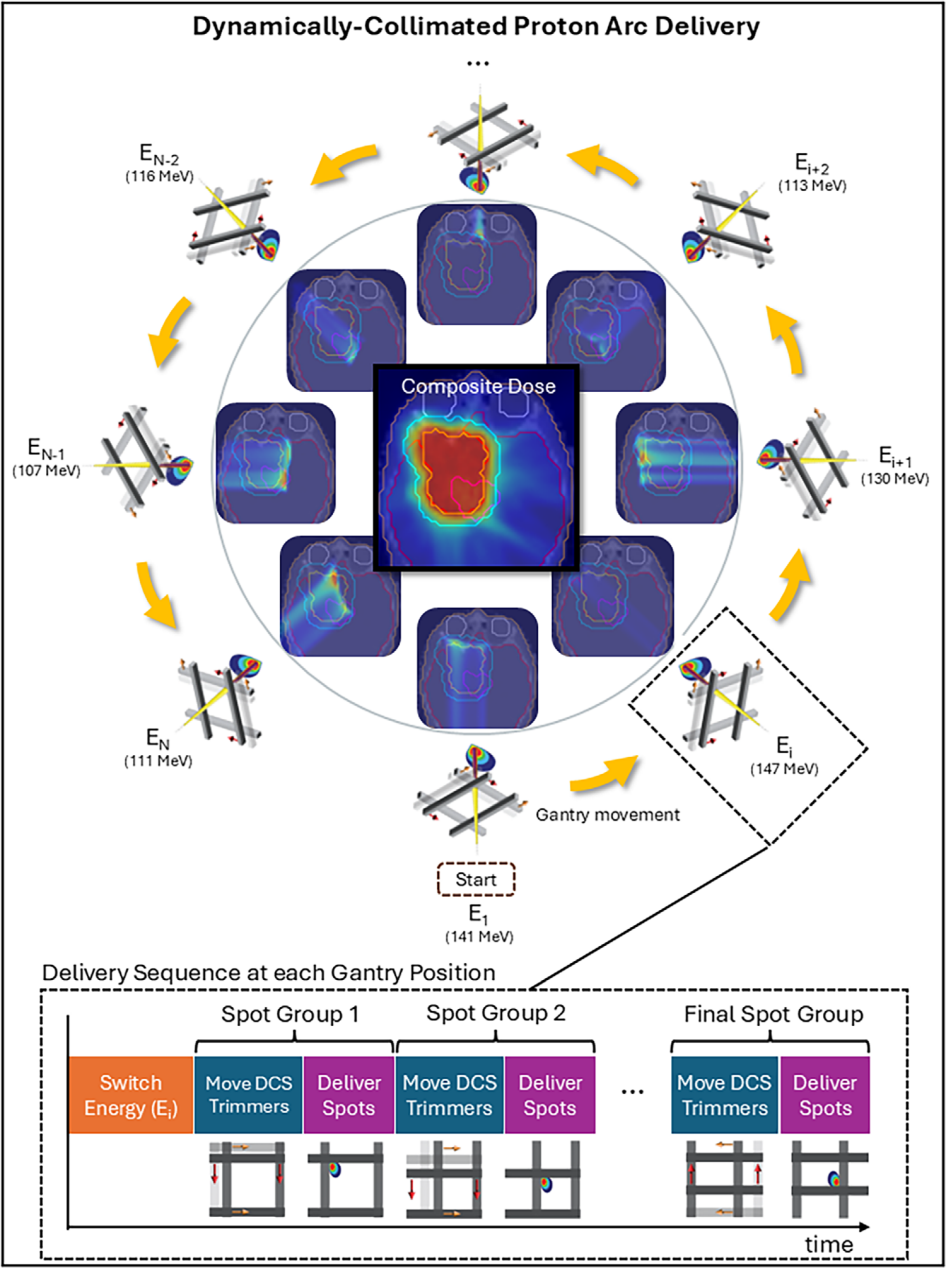
Diagram of the delivery sequence of a DCS‐collimated discrete arc made up of N gantry angles. Yellow arrows indicate the time spent moving the gantry between planned gantry positions. The inscribed box shows the sequence of events at each control point starting with energy switching (orange) and a sequential alternating pattern of DCS trimmer positioning (blue) and spot delivery (purple). The colored rectangles only indicate the order in which delivery actions are performed and are not scaled to represent the time spent on each action.

A diagram of DC‐PAT delivery is provided in Figure 2 to indicate the order of events including energy switching, collimation, spot delivery, and gantry movement. The DCS is not currently configured to handle simultaneous movements of trimmers during magnetic scanning or delivery of spots, and it was conservatively assumed in this study that energy switching occurs after reaching a planned step‐and‐shoot gantry position. Thus, all delivery events are strictly sequential.

BDT estimates of IMPT plans include time for manual intervention between beam arrangements commonly performed in clinical practice, such as patient alignment adjustments, re‐imaging, and final readiness checks. Similar to the methodology described by Wuyckens et al. (2024), a time penalty was included using a sum of 30 s between beams with an additional 120 s for couch‐kicks of non‐coplanar beams.^32^

### 2.3 Delivery time reduction techniques

The three post‐processing techniques of the CSG algorithm, described below, were applied to the baseline plans aimed at improving delivery efficiency without substantially compromising plan quality.

#### 2.3.1 Cut

The weight of each control point was calculated as the sum of MU for all delivered spots. Weights of all control points were normalized to the average control point weight across the entire baseline plan. A threshold of 30% was established, and any control point with a normalized weight below this threshold was cut from the plan. Subsequently, the weights of all remaining spots were reoptimized to maintain dose objectives.

#### 2.3.2 Sort

**FIGURE 3:**
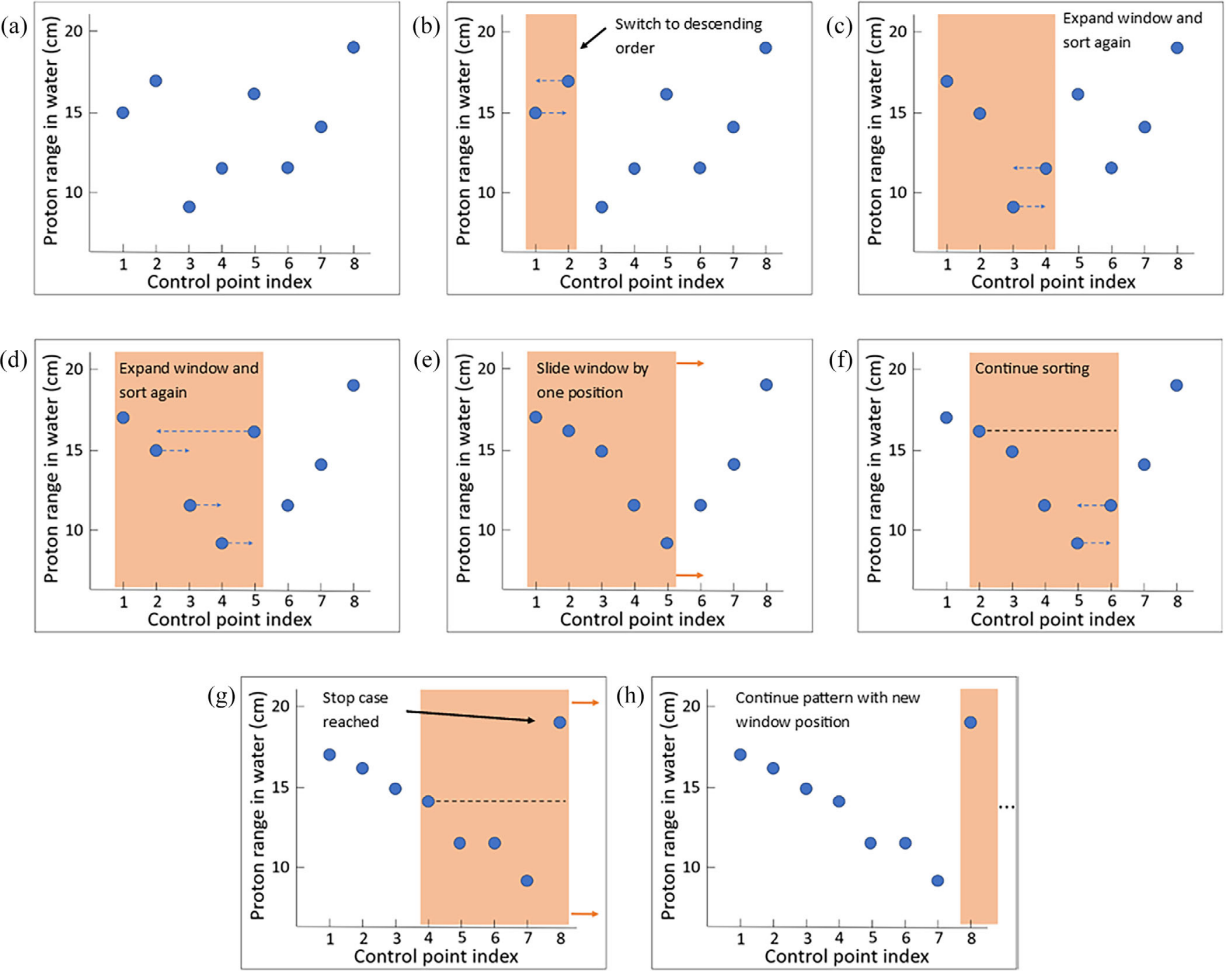
A visual depiction of the sliding window energy layer sorting (SWELS) algorithm sorting the first eight control points in the Patient A baseline plan. A sliding window (orange shading) grows to a predefined size (in this case, five control points contained within 15°). Within the window, energy layers are sorted in descending order, blue arrows in (b), (c), (d), and (f) indicate the new control point energy layers will be re‐mapped to. When sorting is finished, the window slides over one index, as indicated by the orange arrows in (e). This pattern continues until the stop condition is satisfied which is when the proton range of the energy layer in the last index of the window is higher than the first index (g), at which point, the window slides such that the last index becomes new first index (h). This is repeated until all energy layers in the plan are sorted.

The Sliding‐Window Energy Layer Sorting (SWELS) algorithm was developed to reorder energy layers to promote more high‐to‐low energy transitions. This algorithm reorders energy layers within a window of consecutive control points, ensuring only high‐to‐low transitions within that segment. After adjusting the energy layers, the window advances to the next set of control points, maintaining its size. For instance, after sorting control points 1–5, the algorithm proceeds to sort control points 2–6. Because the plan is limited to a single energy layer per control point, this approach effectively applies small changes to the beam angle used for previously optimized control points. The sorting continues until the proton range at the last index of the window exceeds that of the first index, prompting the window to move forward and reassess a new set of control points. This ensures that no energy layer is remapped to a control point location further away than the specified window size. The aggressiveness of SWELS is controlled by the size of the sliding window, which has units of degrees, referring to the subsection of an arc in which control points will be considered for sorting. The maximum number of consecutive control points which can be sorted in a window is found by dividing its size by the resolution of the arc plan. A window size of 15° in a 2.8° resolution arc would amount to a window containing at most five consecutive control points. Figure 3 illustrates this example for the first eight control points in the Patient A baseline plan.

#### 2.3.3 Group

While the baseline plans feature individual trimmer configurations for each spot, an ant colony spot grouping optimization, described elsewhere,^27^ was used to group spots such that a user‐specified number of collimated spots (group size) has a single, shared trimmer configuration. Additionally, the spots with trimmer offsets large enough to be effectively uncollimated are grouped together with the trimmers reassigned to the fully retracted “off” position (6.5 cm from central axis). This reduces the number of unique trimmer configurations in the plan and the amount of time required for trimmers to move between configurations. The mean group size per configuration was progressively increased until efficiency gains plateaued.

### 2.4 Implementation

Each component of CSG was applied to modify the baseline plans across different patients and individually evaluated for its impact on BDT reduction and plan quality. To assess the effectiveness of spot grouping in reducing BDT, the group size parameter was gradually increased until efficiency gains plateaued. The baseline plans were also modified using SWELS with window sizes of 10°, 25°, and 40°. An alternative iterative approach could be employed to the “cut” component of CSG to progressively remove control points until the plan quality falls below a predetermined acceptable level.^21^ However, to limit the number of variables in this study, control point removal was evaluated using a fixed threshold of 30% as discussed in Section II.C.1.

**FIGURE 4:**
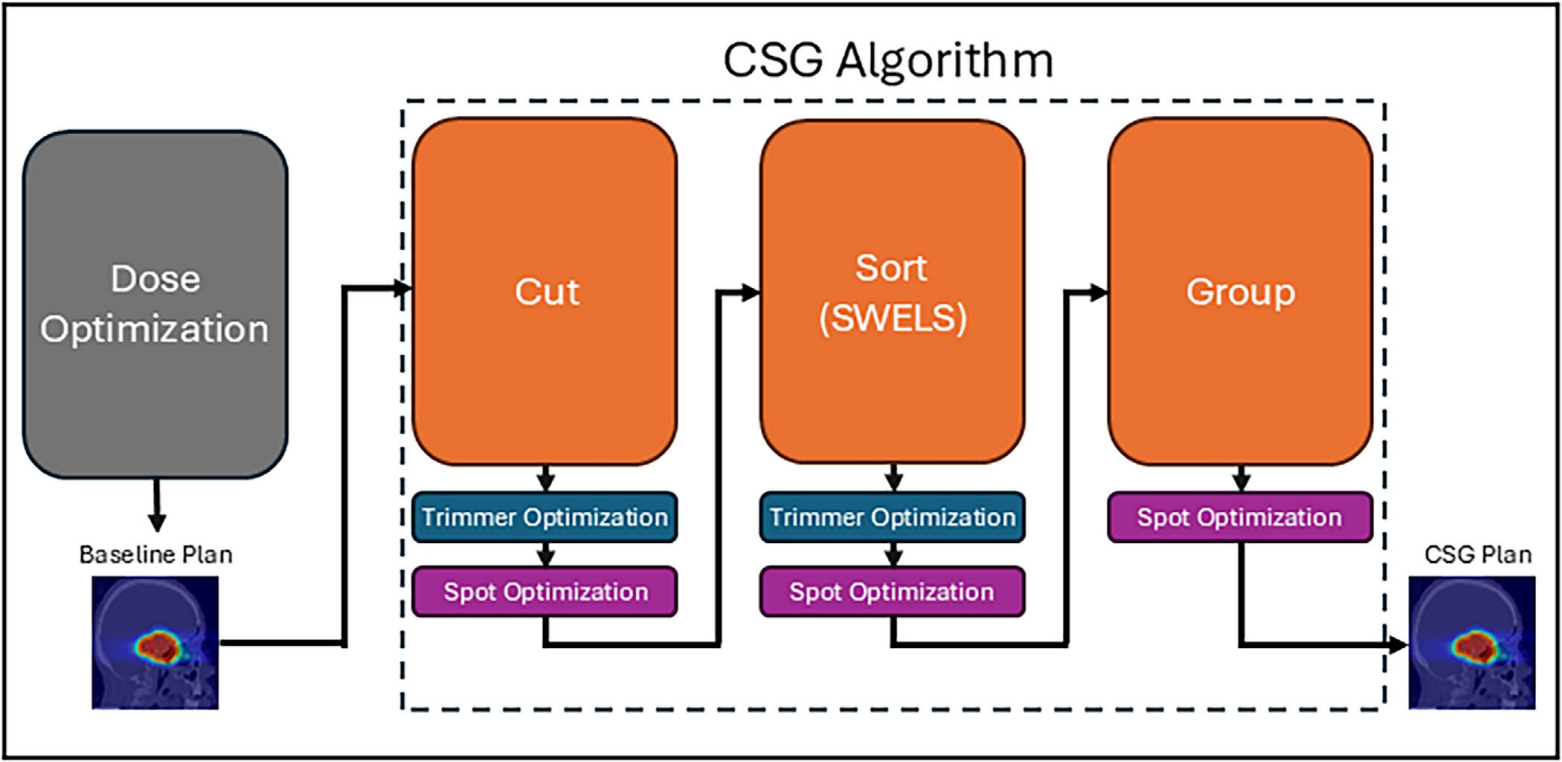
Flowchart of the CSG algorithm. A genetic dose optimizer is used to create the baseline plan which then is processed with cut, sort, and group operations. Prior to moving to the next CSG operation, trimmers and spots are reoptimized to recover dose objectives. Trimmer positions are not reoptimized after “Group” as the spot grouping algorithm discovers more efficient trimmer positions.

After establishing the optimal parameters for each CSG component, they were combined to optimize the baseline plans for each patient, allowing us to evaluate the cumulative benefits and efficiency gains achievable through the implementation of the full CSG algorithm. A flowchart of the CSG algorithm is provided in Figure 4 to illustrate the order in which components of CSG and optimization were implemented.

### 2.5 Evaluation

Unless otherwise specified, all comparisons of the BDT and quality of plans after post‐processing are in reference to the baseline plan from which they are derived. All four components of BDT were examined to assess where time savings occurred. Plans were quantitatively assessed using dose‐volume histogram (DVH) metrics, Homogeneity Index (HI), Conformity Index (CI), and Gradient Index (GI), as defined below^33^, ^34^:

$$HI=\frac{D_{2\%}-D_{98\%}}{D_{p}}$$

$$GI=G\;I_{Paddick}=\frac{V_{50\%}}{V_{100\%\;}}$$

$$CI=C\;I_{Paddick}=\frac{TV_{PIV}}{TV}\;\times \frac{TV_{PIV}}{V_{100\%}}$$

Here, $D_{2\%}$ and $D_{98\%}$ represent the dose to 2% and 98% of the Planning Target Volume (PTV), respectively. $D_{p}$ is the prescription of 50 Gy to the 95% isodose level. $V_{50\%}$ and $V_{100\%\;}$ are the volumes receiving 50% and 100% of the prescription dose, respectively. $TV_{PIV}$ is the target volume receiving the prescription dose or higher, and TV is the target volume. Dose coverage of the PTV was represented with the $\;D_{2\%}$ and $D_{98\%}$ as recommended in ICRU guidelines.^35^ Additionally, the dose to OARs was monitored by calculating the mean dose and $\;D_{2\%}$.

A robustness analysis was conducted for each plan by introducing small perturbations to plan parameters, reflecting expected delivery uncertainties, and recalculating the dose to evaluate changes in dose distribution relative to the nominal planned dose. This analysis accounted for range uncertainty, systematically sampled at 3.5% (95th percentile)^36^, ^37^; spot placement uncertainty, randomly sampled per spot (σ = 0.5 mm)^38^, ^39^; and a conservative estimate of gantry uncertainty, uniformly sampled within one degree. To evaluate the uncertainty associated with DCS alignment when mounted to the proton nozzle, new trimmer positions were sampled to simulate a shift in DCS alignment by σ = 0.05 mm and σ = 0.1 mm in the IEC X and Y directions, respectively. Additionally, trimmer position uncertainty was sampled at σ = 0.3 mm. These uncertainties were derived from mechanical characterization measurements of the DCS by Geoghegan et al.^40^ Patient setup uncertainty was not included, as this analysis specifically focuses on uncertainties inherent to the treatment delivery system, which are separate from setup corrections typically managed through image guidance and immobilization. The dose was recalculated with these adjustments 100 times and the DVH of the nominal planned dose was compared to the 100 resulting outcomes. Dose indices of the Clinical Target Volume (CTV) and other relevant structures were compared across baseline and CSG‐derived plans to assess the effect of the post‐processing algorithm on robustness.

## 3 RESULTS

### 3.1 Delivery time

In the baseline plans for Patients A, B, and C, the number of low‐weight control points cut was 14, 20, and 22, respectively, resulting in time savings of around eight percent for each patient. This led to less energy switching time due to fewer energy layers and decreased gantry motion time, as the removal of control points allows the gantry to pass through those positions without stopping for static spot delivery.

**FIGURE 5:**
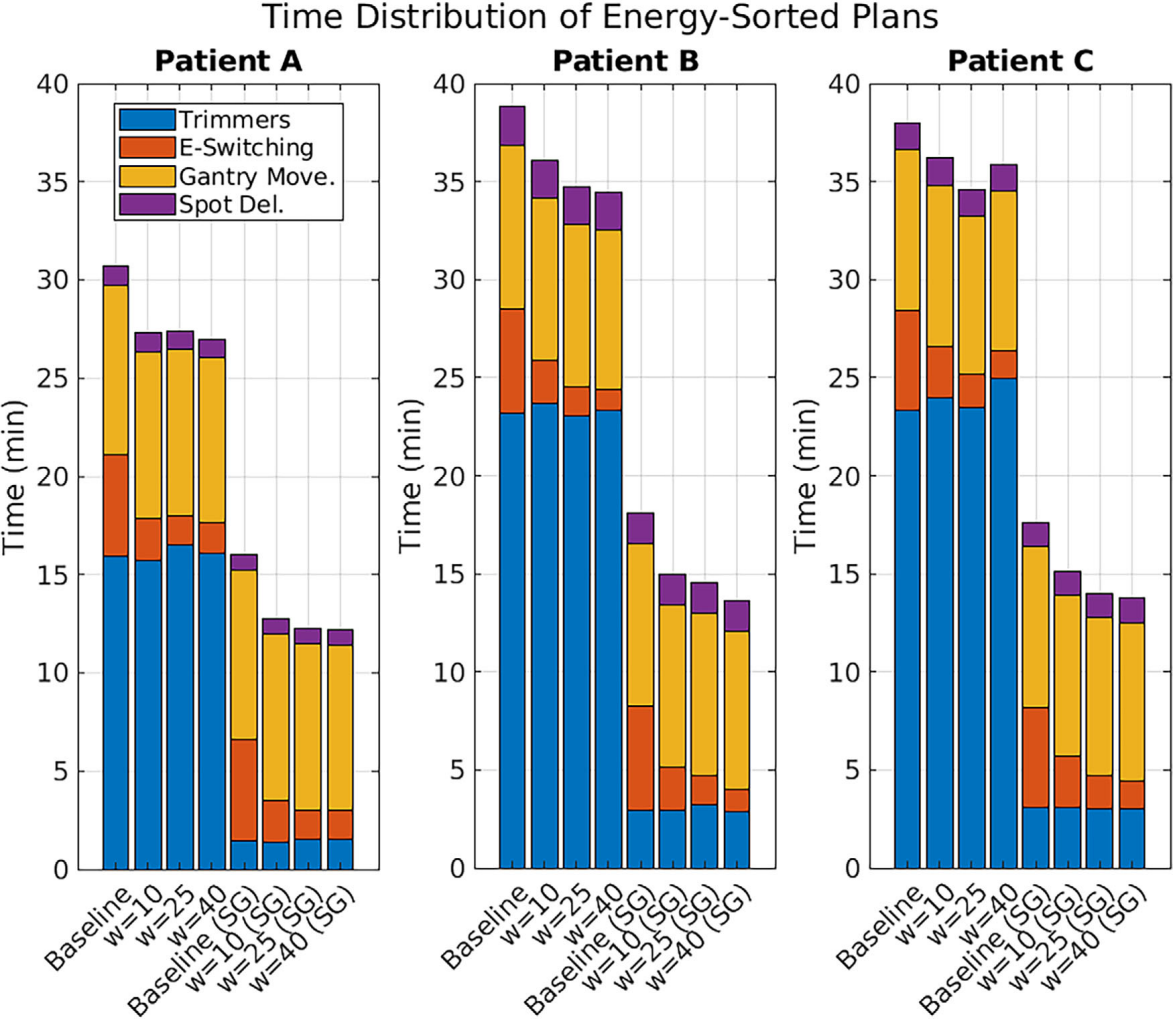
The expected time for trimmer motion (blue), energy switching (orange), gantry motion (yellow), and spot delivery (purple) for the baseline and energy‐sorted plans. The plans with energies sorted with a sliding window of size 10, 25, and 40 degrees are included with spot grouping (SG) and without spot grouping.

There were more than 50 low‐to‐high energy transitions in the baseline plans for each patient. Using SWELS with window sizes of 10, 25, and 40 degrees reduced this number to 21, 15, and 15, respectively, for Patient A. Similarly, energy jumps were reduced to 22, 15, and 11 for Patient B, and 26, 17, and 14 for Patient C. This adjustment reduced energy switching time from more than 300 s to a minimum of 90, 66, and 84 s, for the respective patients. To maintain dose objectives, spot placement and weights were reoptimized, introducing more collimated spots and collimation time, partially offsetting efficiency gains from energy layer sorting. However, combining spot grouping with SWELS mitigated the impact, as shown in Figure 5, demonstrating decreasing delivery times with increasing sliding window sizes for each patient.

**FIGURE 6:**
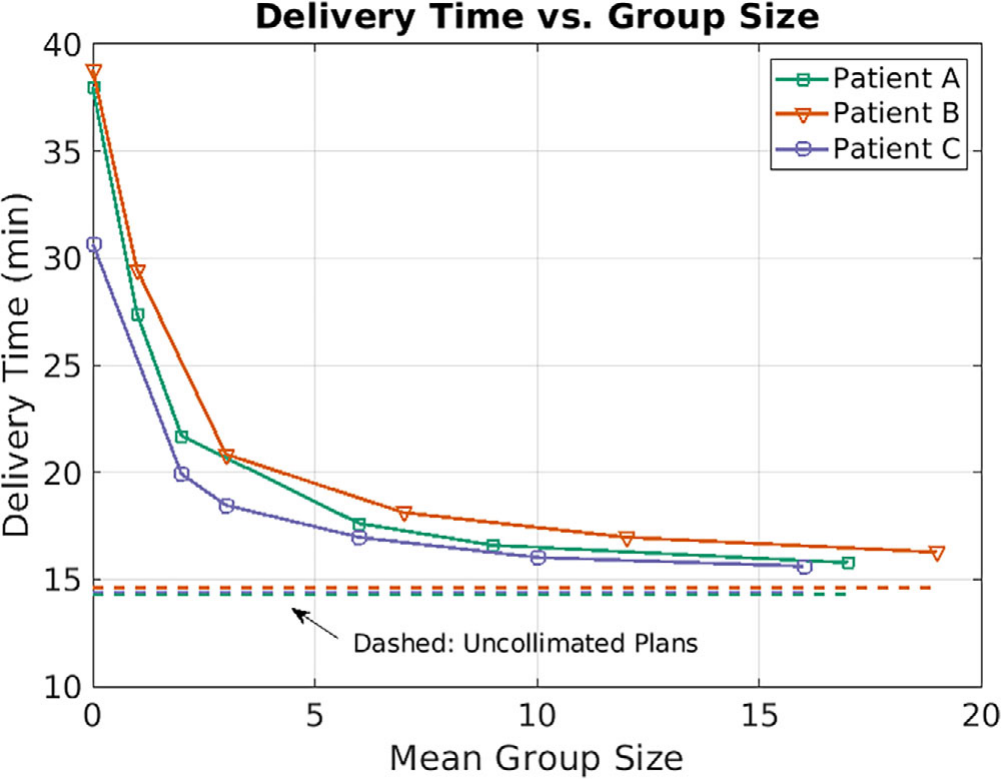
The expected delivery time as a function of the average number of spots in each group of shared trimmer positions. The baseline plans are depicted as having a group size of zero. Plans with a group size of one differ from baseline in that their uncollimated spots are grouped together with trimmers in the “off” position. Dashed lines show the values from the uncollimated plans for comparison.

The ant colony spot grouping algorithm proved very impactful in decreasing the collimation penalty in the collimated arc plans. Increasing the group size initially led to a sharp decrease in BDT, leveling off with diminishing returns as group sizes increased, with the BDT nearing that of an uncollimated plan (Figure 6). For all three patients, the plans with spot grouping with a mean group size of nearly six were between 44% and 53% faster to deliver than baseline plans without grouping. Moreover, these plans were at most 24% slower than the uncollimated baseline plans, while providing improved dose distribution through spot collimation—a relatively modest time penalty for the dosimetric benefits gained. This efficiency is attributed to a nearly tenfold reduction in the number of unique trimmer configurations in the grouped plans compared to the baseline plans for each patient.

The best balance of efficiency gains and plan quality preservation was found when using a window size of 25° and a group size between 6 and 10, depending on the patient. For plans using this configuration, the CSG algorithm reduced expected BDT from 30.7 to 10.8 min (65%), 38.8 to 13.6 min (65%), and 38.0 to 12.5 min (67%) for Patient A, B, and C, respectively.

### 3.2 Plan quality

The plans modified only by removing low‐weight control points (“cut”) were virtually identical to the baseline plans when evaluated using DVH metrics. Likewise, applying the SWELS algorithm (“sort”) in isolation did not result in meaningful changes in dose distribution except for the brainstem's mean dose of Patient A and C. For sliding window sizes of 10, 25, and 40 degrees, this value differed from baseline by 0.4%, 2.4%, and 6.6% and 1.6%, 1.9%, and 1.2%, for Patient A and C, respectively. The “group” component of CSG, when applied alone, resulted in negligible changes in target coverage and only a slight impact on the dose to OARs, even for group sizes greater than 16. The most affected structure, the 10 mm rind surrounding the PTV, experienced only a gradual increase in mean dose as a function of group size, never exceeding two percent of baseline for all patients.

**FIGURE 7:**
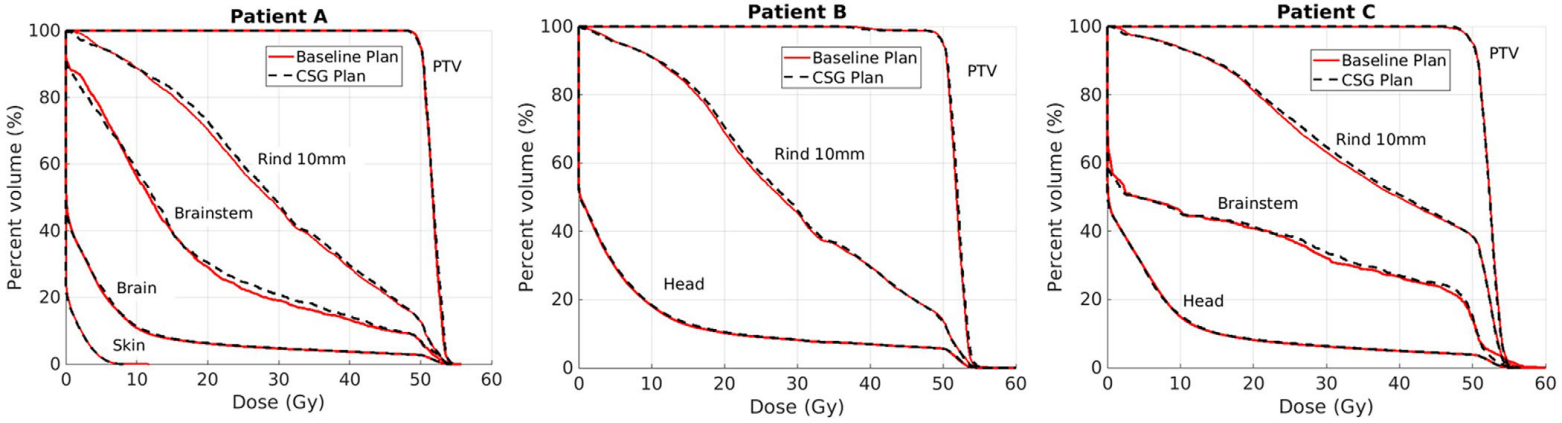
Dose‐volume histograms for the baseline plan (solid, red) and the CSG‐derived plan (dashed, black) for each patient. Each CSG plan included the removal of low‐weight control points, energy layer re‐sorting with a window size of 25°, and a mean spot grouping size of between 6 and 10.

The DVH of each patient's baseline plan was compared to the plans after applying the CSG algorithm (Figure 7). This comparison revealed that the CSG‐derived plans exhibited only marginal changes in dose distribution. The dose coverage of the PTV in each patient's CSG plan was comparable to that of the baseline plan, each achieving a D_2%_ of less than 10% of the prescription and nearly identical target homogeneity.

A summary of the delivery times and plan quality metrics for the plans in this study, both with isolated components and the full CSG algorithm, as well as the IMPT plans, is provided in Table 1. The DC‐PAT plans have increased low‐dose volumes compared to IMPT and higher treatment times overall, largely due to costly gantry movement inherent in discrete arcs. However, these downsides are offset by notably improved dose conformity compared to IMPT, as demonstrated by reductions in GI (18%–38%) and mean dose to the 10 mm Rind (15%–24%).

**TABLE 1:** Summary of delivery times and plan quality metrics for the baseline arc plan, time‐optimized arc plans, a three‐field IMPT plan, and an uncollimated arc plan for each patient.

	CSG Component			Delivery Time (min)					Plan Quality Indices			PTV (Gy)			10 mm Rind (Gy)		Brainstem (Gy)	
	Cut	Sort	Group	Total	DCS/ IMPT^a^	ES^b^	Gantry Move.	Spot Del.	HI	CI	GI	D_98%_	D_50%_	D_2%_	Mean Dose	D_2%_	Mean Dose	D_2%_
**Patient A**	(no modifications)			30.7	15.9	5.2	8.6	1.0	0.09	0.93	2.18	49.5	51.7	53.8	29.4	52.9	17.1	52.3
	✓			28.0	15.8	3.9	7.4	0.9	0.08	0.93	2.15	49.4	51.7	53.5	29.4	52.8	17.0	52.4
		✓		27.4	16.5	1.5	8.4	1.0	0.08	0.93	2.17	49.5	51.8	53.7	29.5	52.9	17.5	52.7
			✓	16.0	1.4	5.2	8.6	0.8	0.08	0.93	2.21	49.4	51.7	53.7	29.7	52.8	17.2	52.3
	✓	✓	✓	10.8	1.4	1.2	7.4	0.8	0.09	0.93	2.21	49.4	51.8	53.8	29.8	53.0	17.8	52.9
	Three‐Field IMPT			7.0	5.0	0.8	0.5	0.6	0.09	0.90	3.00	49.5	51.7	53.8	37.0	52.5	16.1	52.5
	PAT (no modifications)			14.4	0.0	5.2	8.6	0.6	0.09	0.92	2.72	49.3	51.9	53.9	34.3	52.8	19.4	52.4
**Patient B**	(no modifications)			38.8	23.2	5.3	8.4	1.9	0.10	0.93	1.61	49.1	51.9	53.9	29.5	53.4	0.0	0.1
	✓			35.8	22.4	4.0	7.5	1.9	0.10	0.93	1.61	49.1	51.9	53.9	29.4	53.3	0.0	0.1
		✓		34.7	23.0	1.5	8.3	1.9	0.10	0.94	1.63	49.0	52.0	54.1	29.5	53.3	0.0	0.1
			✓	18.1	2.9	5.3	8.3	1.6	0.10	0.94	1.63	49.0	52.0	54.2	29.6	53.3	0.0	0.0
	✓	✓	✓	13.6	3.1	1.3	7.7	1.6	0.10	0.94	1.64	49.0	52.0	54.2	29.7	53.3	0.0	0.0
	Three‐Field IMPT			6.8	4.0	1.0	0.7	1.1	0.13	0.93	1.93	48.9	52.4	55.5	34.2	53.8	0.0	0.1
	PAT (no modifications)			14.6	0.0	5.3	8.3	1.1	0.11	0.93	1.93	48.9	52.1	54.2	34.7	52.9	0.3	1.3
**Patient C**	(no modifications)			38.0	23.3	5.1	8.2	1.4	0.12	0.93	1.86	48.9	52.4	54.9	36.7	54.3	22.9	54.9
	✓			34.8	22.3	4.0	7.2	1.3	0.12	0.94	1.86	48.9	52.4	54.8	36.7	54.2	22.8	54.8
		✓		34.6	23.5	1.7	8.1	1.3	0.12	0.94	1.89	49.0	52.4	54.9	36.8	54.3	23.4	54.0
			✓	17.6	3.1	5.1	8.2	1.2	0.11	0.94	1.91	49.0	52.4	54.7	37.1	54.3	25.2	53.3
	✓	✓	✓	12.5	3.3	1.2	6.8	1.2	0.11	0.94	1.91	49.0	52.4	54.6	37.0	54.2	25.2	53.1
	Three‐Field IMPT			7.2	5.0	0.9	0.3	1.0	0.18	0.87	2.60	45.8	52.4	54.8	42.7	54.1	21.8	55.1
	PAT (no modifications)			14.3	0.0	5.1	8.2	1.0	0.27	0.81	2.18	45.0	52.8	58.5	42.5	57.7	22.6	58.2

^a^ DCS Trimming Time in DC‐PAT plans or Manual Intervention Time for IMPT (e.g., couch kicks).

^b^ Energy Switching.

**FIGURE 8:**
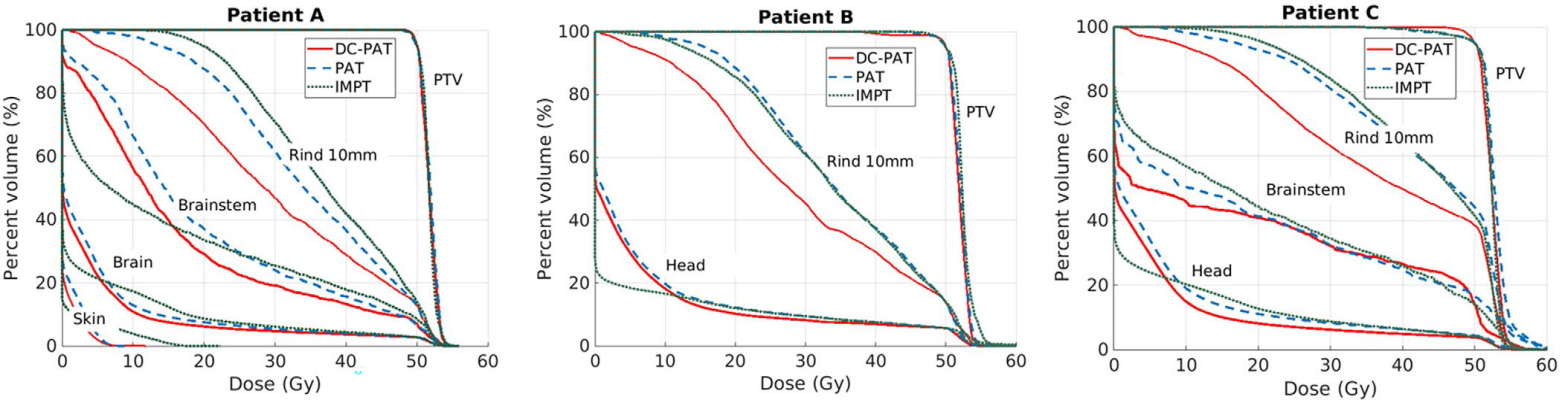
Dose‐volume histograms for the baseline DC‐PAT plan (solid, red), the uncollimated PAT plan (dashed, blue), and the IMPT plan (dotted, green) for each patient.

The DVH plots in Figure 8 compare the baseline DC‐PAT plans with uncollimated plans to show the dosimetric improvement afforded by DCS collimation. Across all three patients, the collimation greatly reduces dose to the 10 mm rind surrounding the target as well as the nearby OARs.

### 3.3 Plan robustness

**FIGURE 9:**
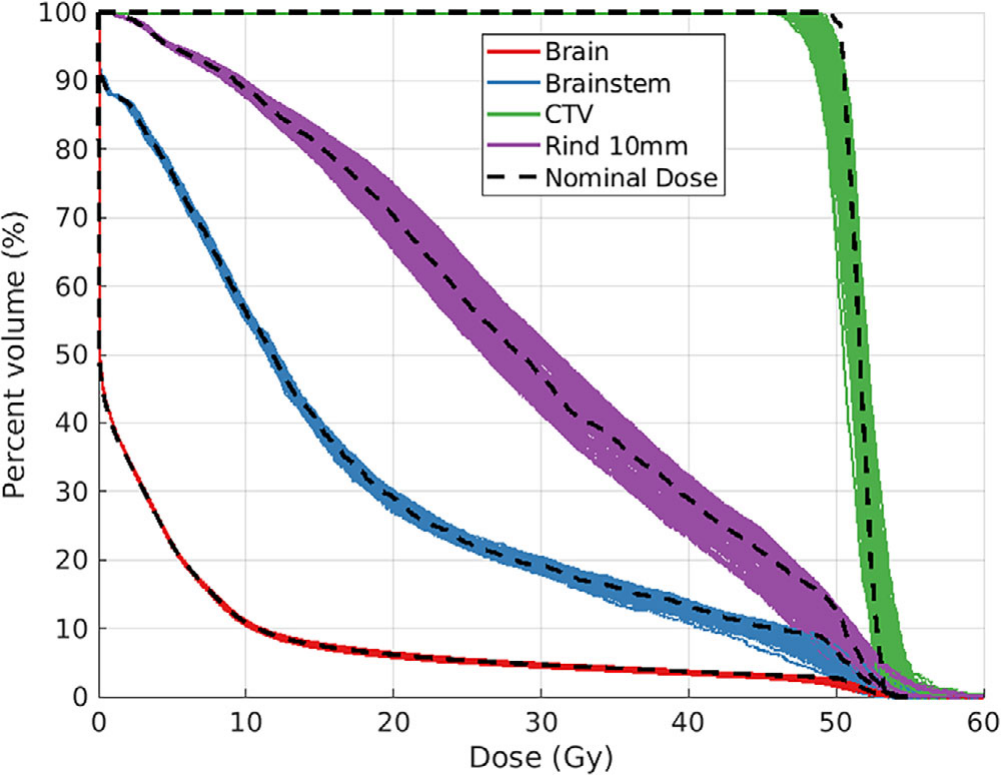
DVH robustness plot for the baseline plan of patient A. Solid colored lines represent the recalculated dose distributions based on sampled uncertainties, while the black dashed line represents the nominal planned dose.

**FIGURE 10:**
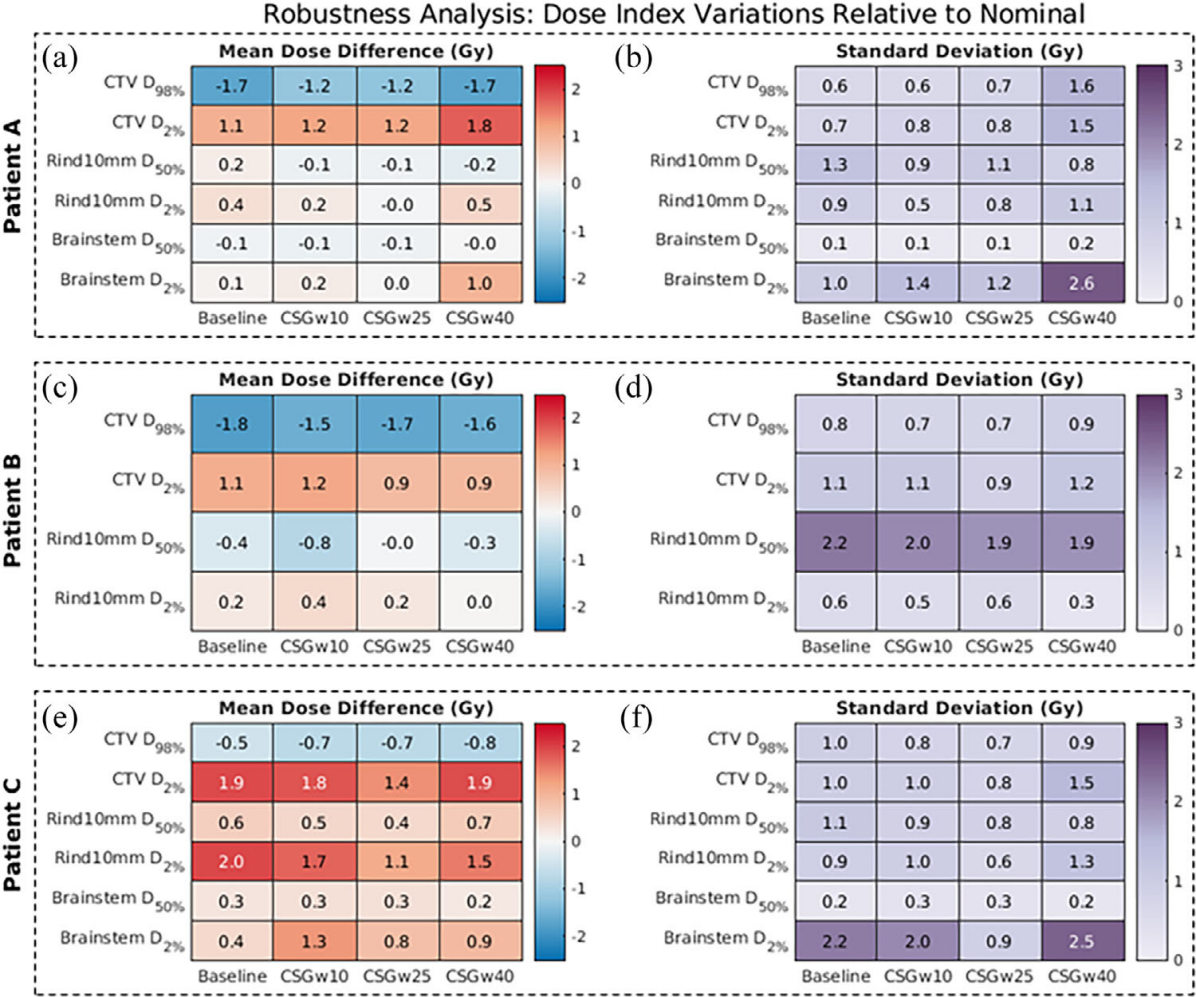
Heatmaps showing dose index variations relative to the nominal distribution. Panels (a), (c), and (e) display the mean dose index differences (Gy), while panels (b), (d), and (f) show the standard deviations of the 100 resampled dose distributions. Rows correspond to relevant anatomical structures, and columns represent different treatment plans. Darker colors in the mean difference maps indicate greater deviations from the nominal dose, whereas darker colors in the standard deviation maps reflect increased variability among the resampled distributions. The first column of each panel shows the dose statistics for the baseline plans, while the remaining three columns represent the CSG plans, differentiated by the sliding window sizes of 10, 25, and 40 degrees.

The DVH robustness plot for the baseline plan of Patient A is shown in Figure 9, where colored lines represent the 100 recalculations of dose after altering plan parameters based on sampled uncertainty. To summarize the uncertainty of baseline plans against CSG‐derived plans across all patients, dose indices for relevant structures were recorded and the averages and standard deviations of each metric for the 100 resampled dose distributions were compared to nominal. The difference is recorded in the heatmaps in Figure 10, where the indices in the plot of Figure 9 correspond to the first columns of Patient A's heatmaps in Figure 10.

In general, the dose indices of CSG‐derived plans do not substantially deviate from nominal compared to baseline plans. There is some notable increase in standard deviation for the CSG plan with the most aggressive sliding window size of 40° for Patient A, seen in the fourth column of Figure 10a. The 40° window size CSG plan of Patient A also shows increased mean dose differences compared to basaeline, as seen in of Figure 10a. However, across all three patients, the CSG algorithm maintains roughly the same robustness as baseline plans when using sliding window sizes of 10 or 20 degrees.

## 4 DISCUSSION

This study demonstrates that post‐processing techniques in the CSG algorithm can be applied to DC‐PAT plans to remove low‐contributing control points, reduce low‐to‐high energy transitions, and decrease the number of unique trimmer configurations with spot grouping to dramatically reduce the expected delivery time with minimal impact on plan quality. The optimal balance of time savings and plan quality was achieved using the SWELS energy sorting window size of 25° and spot grouping with an average group size of 6 to 10, resulting in at least 65% faster delivery for Patients A, B, and C, with total times of 10.8, 13.6, and 12.5 min, respectively. If CSG were applied to a treatment plan intended for a different delivery system than the one used in this study, different parameters could (and should) be used to produce delivery time estimates appropriate for that system.

The SWELS algorithm greatly reduced the number of time‐consuming low‐to‐high energy switches and the corresponding energy switching time using a 10° window size, with diminishing returns for larger windows sizes as the number of energy jumps approach zero. Promoting high‐to‐low energy transitions can result in suboptimal energy layer selection at certain gantry positions. However, this study shows that if the arc plan has sufficient flexibility, re‐optimization of spot weights after energy layer sorting can effectively restore the dose distribution, even for larger window sizes of up to 40°.

This is the first study to quantify the impact of using ant colony optimization for spot grouping in an arc application. When using a spot group size of around six, collimated plans are expected to be less than 25% slower than their uncollimated counterparts—a manageable trade‐off to harness the dosimetric benefits of collimation shown in Figure 8. In baseline plans, each collimated spot was positioned with a 0 mm offset from the beam spot central axis to maximize collimation efficacy. However, when implementing spot grouping, the average trimmer offset increases—as some spots are permitted to be less aggressively collimated in favor of reducing trimmer configuration changes. This results in increased penumbra, explaining the slight increase in mean dose to the 10 mm rind as group size increases. Nevertheless, the overall impact on plan quality from spot grouping remains minimal and is considered a viable trade‐off by producing PAT plans which are fast enough to deliver in a clinical workflow.

This study demonstrated the dosimetric benefits of collimation in PAT by integrating the DCS with the IBA Dedicated Nozzle, which has a relatively small spot size (median: 3.2 mm between 100 and 230 MeV).^41^ Combining collimation with PAT systems that use larger spot sizes, such as the Mevion proton therapy system (Mevion Medical Systems, Littleton, MA, USA), could provide even greater dose sparing of non‐target tissues.

Various investigations into PAT efficiency in the past have used some form of rudimentary BDT estimators. In the simplest form, the number of energy jumps are used as an indicator of plan efficiency. Other studies have used data fitting of electronic log files to predict spot delivery time and magnet scanning time based on spot weight and spot spacing^31^, ^42^ The open‐source ATOM algorithm has been used for BDT estimation for PAT efficiency investigation^32^ however, it is primarily designed to optimize velocity vectors for faster delivery of dynamic PAT.^43^ Prior DCS investigations used trimmer operational parameters such as velocity, acceleration, and jerk, to estimate the extra time required to deliver DCS‐collimated fields due to trimmer movement.^14^, ^40^ This study's approach, using best‐fit curves from experimentally delivered log files to determine spot delivery time as a function of prescribed weight and trimmer travel distance, offers a more realistic and precise prediction of delivery times based on real world data. While using log data for predictive beam delivery is not novel, this work is the first to introduce such an approach for quantifying pauses in spot delivery to allow for DCS trimmer positioning.

Recent studies^20^, ^21^, ^22^, ^23^, ^24^, ^25^, ^44^ from various groups have demonstrated new algorithms for incorporating energy layer sorting and energy reduction during the plan optimization stage to create more efficient PAT plans. A recent study by Wuyckens et al.^45^ reviewed the state‐of‐the‐art ELO‐SPAT^23^ and SPArc^20^ algorithms and compared them to three newly proposed optimization methods and concluded that different optimizers may be better suited for specific clinical cases. This variability makes it difficult to determine which approach is most effective for solving the complex problem of creating efficient and robust arc plans with optimal dose distributions for any given PAT case. The complexity of PAT optimization creates a large solution space, increasing the risk of the optimization process concluding in a local minimum. This might be mitigated with post‐processing adjustments to optimized plans, where further refinements are made to optimize energy layers and reduce switching time. While the concept of energy sorting and reduction is not novel, the unique contribution of this study lies in the sorting logic and post‐processing approach using CSG techniques. These techniques can be applied independently of the treatment planning system or optimization algorithm used to generate the initial plan. They are relatively simple to implement, providing a pathway to more efficient plans without requiring the adoption of new optimizers from the ground up.

A potential weakness of the SWELS algorithm is the possibility for energy layers to sort such that the proton range for a given control point falls outside of the target volume. In these cases, the spot re‐optimization would suppress the weights of these spots, effectively removing them from the plan. However, an alternative approach by Wuyckens et al.^32^ uses a geometry‐based pre‐selection of energy layers in descending energy segments before spot optimization, followed by three post‐processing filtering strategies that are comparable to the “cut” component of CSG. The pre‐arrangement of energy layers based on the anatomical features of the patient avoids the possibility of energy layers being sorted outside the target, at the cost of limiting the initial solution space during dose optimization.

In PAT treatment planning, the decision to use full or partial arcs will need to be carefully considered by the clinical treatment team. It has been suggested that centrally located tumors, such as patient A and C in this study, could more appropriately be treated with a full arc, while a non‐centrally located tumor might benefit from partial arcs^46^ For this study, a full 360° arc was strategically selected for each patient, based on the following considerations:

1. Optimization flexibility: A full arc allows the optimizer to explore all potential gantry solutions without a priori knowledge of the optimal start and stop angles. This provides maximum computational freedom to meet optimization objectives effectively.
2. Conservative BDT estimation: Full arcs, by requiring complete gantry rotation, represent a worst‐case scenario for delivery efficiency. The CSG algorithm demonstrated clinically acceptable delivery times for full arcs, suggesting that partial arcs would likely be at least as efficient. Thus, full arcs provide an upper bound for delivery times in DC‐PAT plans.
3. Consistent control point resolution: The 360° arc plans feature a consistent 2.8° spacing between gantry angles for each patient. A partial arc approach, with potentially different start/stop angles for each patient, could induce variable control point resolutions, potentially confounding efficiency comparisons.

Delivery times of optimized plans in other recent PAT efficiency investigations, which used dynamic arcs, have varied between 5 and 7 min,^31^, ^32^, ^43^ similar to the BDT of IMPT plans in this study. This is notably faster than the discrete‐arc CSG plans presented here, though without the added benefit of spot collimation. This study conservatively assumed that all delivery actions are independent events. Further advancements in proton arc delivery with DCS collimation could see simultaneous movements of trimmers during spot scanning and delivery, energy switching during gantry movement, and the change from discrete to dynamic arcs—all of which would further increase the efficiency of DC‐PAT plans. Future investigations into dynamic delivery of DCS‐collimated arc plans could reduce gantry movement time, the largest contributor to total BDT of CSG plans, but this falls outside the scope of this study.

For practical implementation of delivering the efficient CSG plans, further integration of the DCS with the IBA control system is needed to enable auto‐sequencing.^46^ This integration would allow for the automatic loading of all arc plan parameters—fields, trimmer positions, and spot groups—rather than manually loading each field individually. Once this process is in place, DC‐PAT plans will be delivered, and actual delivery times will be compared to the estimates provided in this study.

The scope of this study was limited to determining the optimal window size for SWELS and the group size for spot grouping to enhance efficiency using CSG for three cranial patients. The baseline plans were not robustly optimized due to limitations in the treatment planning system. However, this study demonstrates that the application of the CSG post‐processing algorithm did not reduce their inherent robustness when using sliding window sizes of less than 40°. This suggests that when CSG is applied to robustly optimized plans, it stands to reason that the resulting plans will exhibit similar robustness, due to the small changes introduced to the fields by the CSG algorithm and subsequently small changes observed among the CSG‐processed plans in this study. Future research will verify this once robustness features can be integrated into the optimization workflow for DC‐PAT planning, especially for more complex treatment sites. The results of the robustness analysis in this study are limited to assumptions on delivery uncertainty of the cranial cases examined; an independent analysis would need to be performed when extending the CSG algorithm to other treatment sites.

DC‐PAT plans were compared against standard IMPT plans, highlighting the potential of collimated arc delivery to achieve superior dose sparing in tissues adjacent to the target volume. Analysis of the DVH curves for DC‐PAT, PAT, and IMPT (Figure 8) suggests that the observed benefits of DC‐PAT over IMPT are primarily attributable to the dose sparing achieved through collimation, rather than the additional beam angles provided by arc delivery. Although IMPT is already a mature clinical technique, the DC‐PAT and PAT plans investigated here were constrained by currently available collimated arc treatment planning tools. As these tools continue to be developed, further improvements in dose sparing may be possible. Conclusions to the efficacy of DC‐PAT compared to other modalities remain preliminary until a more comprehensive treatment planning study has been completed with a larger patient cohort; this is left for future work when similar levels of confidence in dose optimization are achieved across the indicated modalities. Consequently, the scope of this work is primarily limited to introducing the CSG algorithm and demonstrating the resulting improvement in collimated arc delivery efficiency.

## 5 CONCLUSIONS

This study presents the effectiveness of the CSG algorithm in modifying DCS‐collimated step‐and‐shoot proton arc plans to substantially reduce the expected delivery time to just over 10 min, with minimal changes to plan quality and robustness. This delivery efficiency approaches that of other advanced optimization algorithms, showcasing the potential to achieve highly efficient collimated arc plans suitable for practical use. Enhanced trimmer sequencing enables DCS‐collimated plans with a cost of approximately 3 min or less, while the reductions in time allocated for gantry movement, spot delivery, and energy switching can be applied equally to uncollimated arc planning.

## CONFLICT OF INTEREST STATEMENT

The authors declare no conflicts of interest.
